# *Rheum turkestanicum *rhizomes possess anti-hypertriglyceridemic, but not hypoglycemic or hepatoprotective effect in experimental diabetes 

**Published:** 2017

**Authors:** Mousa-Al-Reza Hadjzadeh, Ziba Rajaei, Esmaeil Khodaei, Maryam Malek, Habib Ghanbari

**Affiliations:** 1 *Neurocognitive Research Center and Department of Physiology, School of Medicine, Mashhad University of Medical Sciences, Mashhad, Iran*; 2 *Department of Physiology, School of Medicine, Isfahan University of Medical Sciences, Isfahan, Iran*; 3 *Pharmacological Research Center of Medicinal Plants, School of Medicine, Mashhad University of Medical Sciences, Mashhad, Iran*; 4 *Neurogenic Inflammation Research Center, School of Medicine, Mashhad University of Medical Sciences, Mashhad, Iran*

**Keywords:** Rheum turkestanicum, Diabetes mellitus, Hyperglycemia, Hypertriglyceridemia, Oxidative stress

## Abstract

**Objective:**

*R* *heum turkestanicum* (*R. turkestanicum*) rhizomes have been used in Iranain traditional medicine as an anti-diabetic agent. The purpose of the present investigation was to evaluate the anti-diabetic and antioxidant activities of *R. turkestanicum *rhizome extract in streptozotocin-induced diabetic rats.

**Materials and Methods:**

Diabetes was induced by a single intraperitoneal injection of 55 mg/kg streptozotocin in male Wistar rats. Diabetic rats received the decoction extract of *R. turkestanicum *rhizomes at the doses of 200, 400 and 600 mg/kg daily by gavage for 3 weeks. Serum glucose and lipid levels were measured in all groups before diabetes induction and at the end of week 3. Oxidative stress was evaluated in the liver by measurement of malondialdehyde levels and total thiol concentration at the end of the experiment.

**Results:**

A significant increase in serum glucose and triglyceride levels was observed in diabetic rats, which was accompanied by increased malondialdehyde levels and decreased total thiol concentration in the liver after 3 weeks. Treatment of diabetic rats with *R. turkestanicum* rhizome extract at the doses of 200, 400 and 600 mg/kg over a 3-week period did not change serum glucose, hepatic malondialdehyde and total thiol levels in diabetic rats. However, treatment with *R. turkestanicum *extract significantly decreased serum triglyceride levels in a dose-dependent manner at the end of the experiment.

**Conclusion:**

*R. turkestanicum* rhizome extract possess anti-hypertriglyceridemic, but not hypoglycemic or hepatoprotective effect in diabetic rats. Therefore, *R. turkestanicum *rhizome should be consumed with more caution by diabetic patients.

## Introduction

Diabetes Mellitus is a complex metabolic disorder characterized by chronic hyperglycemia due to insulin deficiency and/or tissues resistance to insulin resulting in abnormalities in the metabolism of carbohydrates, lipids and proteins (Kuzuya et al., 2002 ▶). Prevalence of diabetes is increasing annually in a way that according to the World Health Organization (WHO), diabetes will be the seventh cause of death by the year 2030 (Maiese, 2015 ▶). Hyperglycemia and hyperlipidemia are the most common features of diabetes that contribute to the development of several diabetic complications at the macrovascular (coronary artery and cerebrovascular diseases) and microvascular levels (neuropathy, nephropathy and retinopathy) (Upendra et al., 2010 ▶). Production of reactive oxygen species (ROS) and the development of oxidative stress are considered to be the key factors in the pathogenesis of diabetes. Hyperglycemia in diabetes results in overproduction of ROS, which in turn leads to structural damages to the liver, kidney, and pancreas (Ozkaya et al., 2011 ▶). In recent years, there has been a great interest in using medicinal plants that modulate hyperglycemia and hyperlipidemia, improve oxidative stress and prevent diabetes-associated complications (Sharma et al., 2015 ▶; Rajaei et al., 2015 ▶; Rajaei et al., 2013 ▶; Hadjzadeh et al., 2015 ▶).

Rhubarb, from the family of polygonaceae, is a perennial plant species spread from North and Central Asia to the other regions (Andic et al., 2009 ▶). Its wild forms that are found in Iran, are locally known as ″Rivas″. The main bioactive components of rhubarb are anthraquinone derivatives including emodin, aloe-emodin, rhein, chrysophanol, physcion, and Danthron. Other constituents such as dianthrones, stilbenes, anthocynins, falvonoids, anthraglycosides, polyphenols, essential oil, organic acids, chromone glycosides and vitamins have also been isolated from rhubarb (Zhang and Liu, 2004 ▶).

Rhubarb has been used in Iranian traditional medicine to treat gastric and liver disorders, jaundice, constipation, headache, kidney and bladder pain, hemorrhoids, ulcer and diarrhea (Hadjzadeh et al., 2013 ▶). Also, rhubarb has anti-tumor, anti-mutagenic (Zhang and Liu, 2004 ▶) and antioxidant activities (Ozturk et al., 2007 ▶; Hu et al., 2010 ▶).

Several experimental studies have shown that some rhubarb species possess anti-diabetic activity (Ozbek et al., 2004 ▶; Radhika et al., 2010 ▶). For instance, it has been reported that the decoction extract of *Rheum ribes* roots (Ozbek et al., 2004 ▶) and ethanolic extract of *Rheum emodi *rhizome (Radhika et al., 2010 ▶) possess anti-hyperglycemic activity in alloxan-induced diabetic animals. Moreover, it has been shown that the stalk and roots of *Rheum ribes* lowers plasma cholesterol levels in animals on a high-cholesterol diet (Hadjzadeh et al., 2004 ▶) and in hypercholestrolemic human subjects (Goel et al., 1997 ▶).

*Rheum turkestanicum* (*R. turkestanicum*) is one of the rhubarb species that grows in Central Asia and North-East of Iran, especially in borderline regions of Iran and Turkmenistan. Traditionally, decoction of the *R. turkestanicum *rhizomes has been used by local people to treat diabetes. The anti-tumor properties of *R. turkestanicum *rhizomes have been recently reported (Shiezadeh et al., 2013 ▶). However, there is no scientific report on the anti-diabetic effect of *R. turkestanicum. *Therefore, the present study was designed to evaluate the possible anti-hyperglycemic, hypolipidemic and antioxidant effects of *R. turkestanicum *rhizome extract in streptozotocin-induced diabetic rats.

## Materials and Methods

**Animals**

Male Wistar rats, weighing 250-300g were housed in an air-conditioned room at 23 ± 2°C with free access to standard pellet diet and tap water, *at libitum*. The Ethics Committee for Animal Experiments of Mashhad University of Medical Sciences, Mashhad, Iran approved the study and all experiments were conducted in accordance with the National Institute of Health Guide for the Care and Use of Laboratory Animals (NIH Publications No. 8023) revised in 1996.

**Decoction extract of** ***Rheum turkestanicum***

The rhizomes of *R. turkestanicum* obtained from borderline regions of Iran and Turkmenistan. The plant was graciously identified by Ferdowsi University herbarium, Mashhad, Iran (Herbarium Accession No. 42082). Dried rhizomes of *R. turkestanicum *were ground to fine powder and then added to boiling water. After half an hour, the suspension was filtered and the solution was given to animals at doses of 200, 400 and 600 mg/kg, by gavage. 

**Experimental protocol**

Diabetes was induced in the overnight fasted male rats by a single intraperitoneal injection of 55 mg/kg streptozotocin (Enzo Life Sciences, USA) (Rajaei et al., 2013 ▶) freshly dissolved in cold distilled water. A blood sample was collected after 3 days of the streptozotocin injection, and the serum glucose levels were measured using a glucometer (Glucocard, Japan). Only those animals with serum glucose higher than 250 mg/dl were considered as diabetics for the experiments. Diabetes was also confirmed by the presence of polyphagia, polydipsia and polyuria. The day on which hyperglycemia was confirmed was designated as day 0. Then, animals were divided into five groups, each with seven rats, as follows: control, diabetics, diabetics treated with *R. turkestanicum* extract at daily doses of 200, 400 and 600 mg/kg. The animals received the extract by gavage since day 0 for 3 weeks. Changes in body weight, food consumption and water intake were recorded during the experiment period. 

For blood sampling, rats were fasted overnight and blood samples were collected from retro-orbital plexus before diabetes induction (week 0) and at the end of week 3. Blood was allowed to clot and serum was separated by centrifugation at 3500 rpm for 10 min. Serum was then used for estimation of the glucose and lipid levels. At the end of the experiment, the animals were sacrificed and the liver were dissected out, washed immediately in ice-cold saline, and homogenized in KCl solution by a homogenizer (Heidolph).

**Biochemical assays**

Serum levels of glucose, triglycerides and total cholesterol were determined, according to the manufacturer’s instructions, by enzymatic colorimetric methods using commercially available kits (Pars Azmun, Tehran, Iran) by a biochemistry analyzer (Convergys 100, Germany). 

Lipid peroxidation level in the liver was measured as malondialdehyde (MDA) which is the end product of lipid peroxidation, and reacts with thiobarbituric acid to produce a red complex with a peak absorbance at 535 nm. A mixture of tricholoroacetic acid, thiobarbituric acid and HCl was added to 1ml of homogenate, and the mixture was heated for 45 min in a boiling water bath. After cooling and centrifugation at 1000 rpm for 10 min, the absorbance was measured at 535 nm. The concentration of MDA was calculated using the following formula: C (M)=A ⁄1.65×10^5 ^(Sharma et al., 2006 ▶).

Total sulfhydryl (SH) groups were measured using DTNB (2,2´-dinitro-5,5´-dithiodibenzoic acid) as the reagent. This reagent reacts with SH groups to produce a yellow complex with a peak absorbance at 412 nm. Briefly, 1ml Tris-EDTA buffer (pH=8.6) was added to 50 μl homogenate in 2 ml cuvettes and sample absorbance was read at 412 nm against Tris-EDTA buffer alone (A1). Then, 20 μl DTNB reagent (10 mM in methanol) was added to the mixture and, after 15 min, the sample absorbance was read again (A2). The absorbance of DTNB reagent was also read as blank (B). Total thiol concentration (mM) was calculated as follows: Total thiol concentration (mM) = (A2-A1-B) × 1.07/0.05 × 13.6 (Ellman, 1959 ▶).

**Statistical analysis**

The data were expressed as mean ± SEM. Statistical analysis was carried out using one-way ANOVA followed by Tukey *post-hoc* test. A p*<*0.05 was considered as statistical significant.

## Results

**Serum glucose levels**

**Figure 1:**
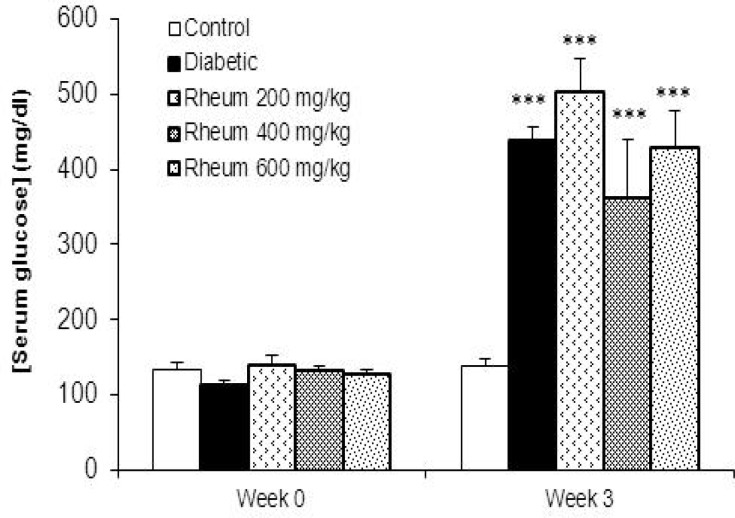
Serum glucose levels (mean ± SEM) in the control, diabetic and diabetic rats treated with the *R. turkestanicum* extract at the doses of 200, 400 and 600 mg/kg at week 0 (before diabetes induction) and at the end of week 3. ***p<0.001 vs. control group

As shown in Figure 1, there were no significant differences in serum glucose levels among animals in the experimental groups at week 0 (before diabetes induction). However, diabetic rats showed a significant increase in serum glucose compared to control rats at the end of week 3 (p<0.001, Figure 1). Treatment of diabetic rats for 3 weeks with *R. turkestanicum* extract at the doses of 200, 400 and 600 mg/kg did not change the serum glucose in comparison with diabetic rats (Figure 1).

**Serum triglyceride levels**

**Figure 2:**
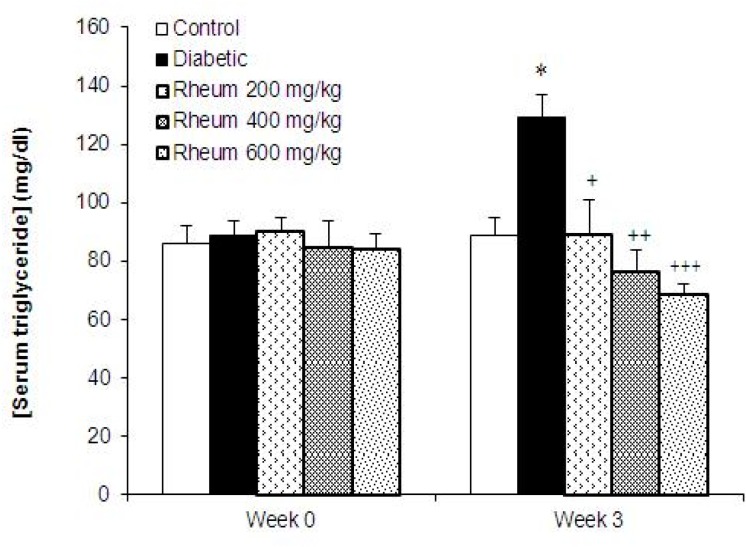
Serum triglyceride levels (mean ± SEM) in the control, diabetic and diabetic rats treated with *R. turkestanicum *extract at the doses of 200, 400 and 600 mg/kg at week 0 (before diabetes induction) and at the end of week 3. *p<0.05 vs. control group, +p<0.05, ++p<0.01, +++p<0.001 vs. diabetic group

The levels of triglyceride were significantly increased in diabetic group compared to control group at week 3 (p<0.05, Figure 2). Treatment of diabetic rats with *R. turkestanicum *extract at the doses of 200, 400 and 600 mg/kg significantly and dose-dependently decreased the triglyceride levels as compared to untreated diabetic rats at the end of week 3 (p<0.05, p<0.01, p<0.001, respectively, Figure 2).

**Serum total cholesterol levels**

**Figure 3:**
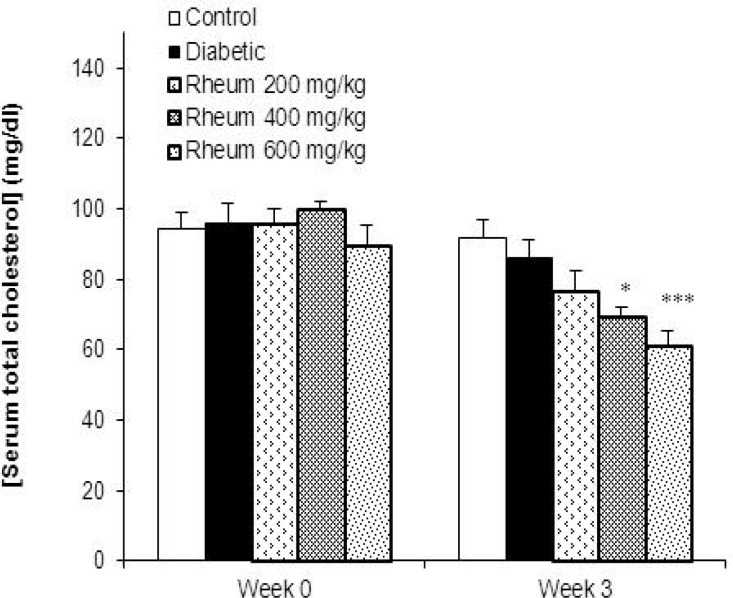
Serum total cholesterol levels (mean ± SEM) in the control, diabetic and diabetic rats treated with the *R. turkestanicum* extract at the doses of 200, 400 and 600 mg/kg at week 0 (before diabetes induction) and at the end of week 3. *p<0.01 and ***p< 0.001 vs. control group

Diabetes induction for 3 week did not change the total cholesterol levels in diabetic animals in comparison with controls. However, treatment of diabetic rats with *R. turkestanicum* extract at the doses of 400 and 600 mg/kg significantly decreased cholesterol levels as compared to control group (p<0.01, p<0.001, respectively) (Figure 3). 

**Malondialdehyde levels in the liver**

**Figure 4:**
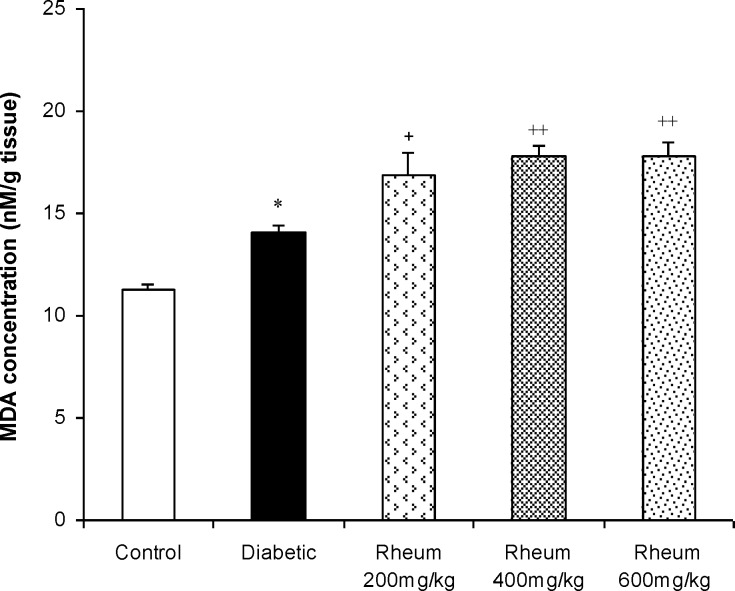
Malondialdehyde (MDA) levels (mean ± SEM) in the liver of the control, diabetic, and diabetic rats treated with *R. turkestanicum* extract at the doses of 200, 400, and 600 mg/kg at the end of week 3.*p<0.05 vs. control group and +p<0.05 and ++p<0.01 vs. diabetic group

MDA levels, an index of lipid peroxidation, in the liver of the control and experimental groups of rats are shown in Figure 4. A significant increase in the levels of MDA in the liver of streptozotocin-induced diabetic rats was found (p<0.05, Figure 4). Treatment of diabetic rats with *R. turkestanicum* extract at the doses of 200, 400 and 600 mg/kg even more significantly increased the MDA levels compared to control group (p<0.01, p<0.001 and p<0.001, respectively) (Figure 4).

**Total thiol concentration in the liver**

**Figure 5:**
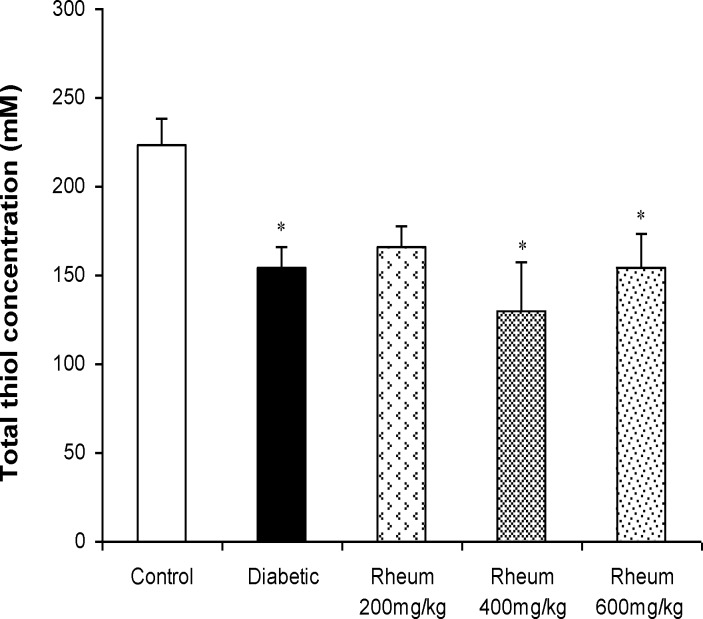
Total thiol concentrations (mean ± SEM) in the liver of the control, diabetic, and diabetic rats treated with *R. turkestanicum* extract at the doses of 200, 400, and 600 mg/kg at the end of week 3. *p<0.05 vs. control group

A significant decrease in the total thiol concentration in the liver of diabetic rats was observed when compared to control rats (p<0.05). Administration of *R. turkestanicum* extract at the doses of 200, 400 and 600 mg/kg did not change the total thiol concentration in the liver of diabetic rats (Figure 5).

## Discussion

Diabetes mellitus is a metabolic disorder affecting carbohydrate, fat, and protein metabolism. Diabetes is characterized by hyperglycemia, hyperlipidemia and oxidative stress followed by dysfunction of many organs in the later stages (Kuzuya et al., 2002 ▶; Upendra et al., 2010 ▶). Streptozotocin diabetic model is the most widely used method to induce diabetes comparable to human diabetes. Diabetogenic effect of streptozotocin is due to the excessive production of ROS, which leads to cytotoxicity in pancreatic *β*-cells (Szkudelski, 2001 ▶). This cytotoxic compound specially enters the β-cells via glucose transporter and induces the DNA strand breakage in β-cells causing decrease in insulin release (Szkudelski, 2001 ▶; Kumar et al., 2013 ▶) and subsequent increase in blood glucose levels. Streptozotocin administration generally causes destruction of β-cells after three days in rats (Adeghate and Ponery, 2002 ▶). Pancreatic β-cells are particularly sensitive to damage induced by free radicals and nitric oxide because of the low levels of free radical scavenging enzymes in this tissue (Spinas, 1999 ▶). In the present study, administration of streptozotocin induced a diabetic state confirmed by high blood glucose levels. In addition, daily oral administration of *R. turkestanicum *rhizome extract (200, 400 and 600 mg/kg) for 3 weeks did not decrease the blood glucose levels in diabetic rats. This means that the *R. turkestanicum *extract was not able to scavenge free radicals due to oxidative stress and could not protect pancreatic β-cells. To our knowledge, this is the first study to investigate the effect of *R. turkestanicum *extract on streptozotocin-induced diabetes in a rat model. 

Hyperglycemia is a well-known causative factor for elevation of free radical levels, which can lead to increased lipid peroxidation and alter antioxidant defence (Balasubashini et al., 2004 ▶). The resulting oxidative stress leads to oxidative damage in many organs, including the liver (Kakkar et al., 1998 ▶). Increased lipid peroxidation impairs the membrane function by decreasing membrane fluidity and changing the activity of membrane enzymes and receptors (Arulselvan and Subramanian, 2007 ▶). Liver is an important metabolic organ involved in glucose and lipid homeostasis. Oxidative damage in the liver may disturb glucose and lipid profile. In our study, we observed a significant increase in lipid peroxidation (MDA levels) and decrease in total thiol concentration (non-enzymatic defence potential) in the liver of diabetic rats, which is consistent with previous studies (Rajaei et al., 2013 ▶; Kumar et al., 2013 ▶). Our results also showed that treatment with *R. turkestanicum* rhizome extract, at all studied doses, did not change lipid peroxidation and total thiol concentration in hepatic tissues of diabetic rats. These findings reconfirm lack of antioxidant activity of *R. turkestanicum *extract in diabetic conditions.

Although our results did not show anti-diabetic and antioxidant effects for *R. turkestanicum* rhizome extract on streptozotocin-induced diabetes, the hypoglycemic activity of some *Rheum* species has already been reported in diabetic models (Ozbek et al., 2004 ▶; Radhika et al., 2010 ▶). For instance, Radhika et al. (2010) ▶ reported that oral administration of ethanolic extract of *R. emodi* rhizome at the dose of 250 mg/kg for 30 days resulted in a reduction in blood glucose level in alloxan-induced diabetic rats. Chen and Wang (2010) ▶ have also reported the hypoglycemic and antioxidant effects of *Rheum franzenbachii* root extract in streptozotocin-induced diabetic rats. They found that repeated oral administration of ethanol extract (125, 250, and 500 mg/kg) for 14 days produced a significant fall in plasma glucose level and MDA, while elevated the reduced glutathione levels and superoxide dismutase and catalase activities in diabetic rats. This discrepancy among these studies and our results could be related to the *R. turkestanicum* species and differences in chemical composition among the species. This is confirmed by a study which has reported that there are significant variations in chemical composition among *Rheum ribes *samples collected from six different regions in Eastern Anatolia (Andic et al., 2009 ▶).

Diabetes is also linked with abnormal lipid metabolism that is considered a major risk factor for the development of cardiovascular complications (Saltiel and Kahn, 2001 ▶; Bansal et al., 2012 ▶). Hypertriglyceridemia has been reported to occur in streptozotocin diabetic rats (Sharma et al., 1997 ▶). Under normal conditions, insulin activates lipoprotein lipase which hydrolyzes triglycerides (Taskinen, 1987 ▶). However, in diabetic conditions, it fails to activate the enzyme, resulting in hypertriglyceridemia. In our experiment, increased levels of serum triglycerides were observed after 3 weeks of diabetes, which is in accordance with previous studies. Moreover, repeated administration of rhizome extract of *R. Turkestanicum *for 3 weeks produced a significant decrease in serum triglycerides in streptozotocin diabetic rats. Previously, it has been reported that the stalk and roots of *Rheum ribes *reduced plasma cholesterol levels in animals fed with a high-cholesterol diet (Hadjzadeh et al., 2004 ▶) and in hypercholestrolemic human subjects (Kakkar et al., 1998 ▶). However, to our best of knowledge, this is the first study reporting the anti-hypertriglyceridemic activity of *R. turkestanicum* rhizome in a diabetes model. Phytochemical analysis of rhubarb has revealed the presence of anthraquinone derivatives such as emodin, dianthrones, stilbenes, anthocynins, falvonoids and vitamins (Zhang and Liu, 2004 ▶). Some studies have also shown the effectiveness of flavonoids on dyslipidemia (Coskun et al., 2005 ▶; Anila and Vijayalakshmi, 2002 ▶). Moreover, it has been reported that treatment with emodin, an anthraquinone, for 3 weeks improves lipid profile in diabetic mice (Xue et al., 2010 ▶). Therefore, the observed hypolipidemic activity of *R. turkestanicum* extract could be attributed to the presence of emodin and flavonoids in the extract. 

Our findings demonstrated that the rhizome extract of *R. turkestanicum *possesses anti-hypertriglyceridemic, but not hypoglycemic or hepatoprotective effect in streptozotocin-induced diabetes. More studies are needed to clarify the toxicity of the extract and its effects on hematological, biochemical and histological parameters. Collectively, this study suggests that *R. turkestanicum *rhizome extract should be consumed with more caution until further assurance is given for its safety. 
